# Patient-Reported Epigastric Symptom Dynamics During Trimebutine Maleate Treatment: A Post-Marketing Study

**DOI:** 10.3390/medicina62030499

**Published:** 2026-03-07

**Authors:** Svetoslav Stoev, Vesselina Yanachkova, Elina Petkova-Gueorguieva, Stanislav Gueorguiev, Violeta Getova-Kolarova, Hristina Lebanova, Vasil Koynarski

**Affiliations:** 1 Department of Pharmaceutical Sciences and Social Pharmacy, Faculty of Pharmacy, Medical University-Pleven, 5800 Pleven, Bulgaria; 2 Research Institute, Medical University-Pleven, 5800 Pleven, Bulgaria; 3 Department of Endocrinology, Specialized Hospital for Active Treatment of Obstetrics and Gynecology Dr. Shterev, 1330 Sofia, Bulgaria; 4 Department of Health Management and Health Economics, Faculty of Public Health, Medical University of Sofia, 1000 Sofia, Bulgaria; 5 Department of Pharmaceutical Sciences, Faculty of Pharmacy, Medical University Plovdiv, 4002 Plovdiv, Bulgaria; 6 Department “Organization and Economics of Pharmacy”, Faculty of Pharmacy, Medical University of Sofia, 1000 Sofia, Bulgaria; 7 VITA Multiprofile Hospital for Active Treatment, 1407 Sofia, Bulgaria

**Keywords:** trimebutine maleate, patient-reported outcomes, functional gastrointestinal disorders

## Abstract

*Background and Objectives:* This prospective, non-interventional, multicenter study aims to assess the therapeutic effectiveness of trimebutine maleate in patients with functional gastrointestinal disorders (FGIDs) by patient-reported outcomes. *Materials and Methods:* The study encompassed 2501 patients from 50 clinical sites diagnosed with irritable bowel syndrome, functional dyspepsia, or other functional gastrointestinal diseases. A validated questionnaire, an adapted version of the Izumo scale, was employed for self-assessment of symptoms and quality of life related to gastrointestinal issues. *Results:* The findings indicate a significant decrease in symptom severity, encompassing epigastric pain, dyspeptic symptoms, and defecation difficulties, as early as the second visit, with mean pain scores declining from 2.65 to 0.46 (*p* < 0.001). *Conclusions:* The data validate trimebutine’s profile as a well-tolerated and efficacious therapy for patients with IBS in real-world clinical practice in Bulgaria. The findings endorse the prospective inclusion of trimebutine as a treatment option in forthcoming clinical guidelines.

## 1. Introduction

Functional gastrointestinal disorders (FGIDs) comprise a category of illnesses defined by persistent or recurrent symptoms lacking identifiable biological, biochemical, or metabolic origins. The most prevalent among these are irritable bowel syndrome (IBS), functional dyspepsia, and gastroesophageal reflux disease (GERD). While these illnesses do not correlate with heightened mortality, they impose a considerable burden on patients due to persistent symptoms, diminished daily functioning, and a decline in health-related quality of life [1]. Consequently, conventional clinical or laboratory metrics frequently do not accurately reflect the disease’s actual effect on individuals’ well-being. In this setting, patient-reported outcomes (PROs) have become a pivotal element of clinical assessment and therapy evaluation. Their significance is especially evident in FGIDs, since the disease burden is mostly dictated by subjective symptom assessment rather than quantifiable physiological anomalies.

In recent years, the systematic integration of patient-reported outcomes (PROs) into gastroenterological research and clinical practice has received heightened regulatory and scientific acknowledgment. Regulatory guidance documents have underscored the necessity of well-defined Patient-Reported Outcome (PRO) instruments to substantiate claims of symptomatic benefit, while clinical research frameworks are progressively incorporating PRO measures to record patients’ perspectives on symptom severity and treatment efficacy. In addition, prior studies have shown that PRO measures exhibit the strongest correlation with the severity of abdominal pain, which is the primary symptom affecting patients’ overall evaluation of disease severity in IBS and related conditions [2]. Thus, the utilization of standardized, validated Patient-Reported Outcome measures has emerged as a recognized and methodologically robust tool for evaluating treatment outcomes in functional gastrointestinal diseases [1,2,3].

In addition to being methodologically sound, patient-reported outcome instruments are increasingly being considered as an essential component of international clinical trial designs and regulatory evaluation frameworks, especially in functional gastrointestinal disorders where the main therapeutic goal is symptom relief [4].

The pharmacological treatment of FGIDs frequently focuses on abnormalities in gastrointestinal motility and visceral sensitivity, regarded as fundamental pathophysiological mechanisms that contribute to symptom development. The management of FGIDs frequently involves pharmacotherapy with prokinetic agents, with trimebutine distinguished by its intricate pharmacological mechanism and extensive therapeutic efficacy in addressing both hypermotility and hypomotility disorders of the gastrointestinal tract. In this context, trimebutine maleate represents a rational therapeutic option for the treatment of functional gastrointestinal disorders due to its role as a modulator of gastrointestinal motility [5] and visceral sensitivity, while its practical efficacy largely requires evaluation through patient-reported outcomes. Trimebutine is a partial agonist of peripheral μ-, κ-, and δ-opioid receptors, regulating gastrointestinal motility by influencing the interstitial cells of Cajal [5]. Research indicates that it alleviates abdominal discomfort, modulates intestinal peristalsis, and facilitates gastrointestinal transit in individuals with IBS [6,7]. Despite its prevalent application in standard clinical practice, data regarding patient-reported outcomes associated with trimebutine is scarce, especially within Eastern European demographics. Considering the pivotal significance of symptom perception and quality of life in functional gastrointestinal diseases, assessing therapy efficacy from the patient’s viewpoint constitutes a significant unmet necessity. Despite randomized controlled trials establishing their pharmacological and therapeutic efficacy, there is a paucity of real-world post-marketing evidence, particularly from those analyzing the long-term dynamics of patient-reported symptoms. Multicenter observational data from standard practice may yield significant insights into therapy efficacy, external validity, and patient-perceived advantages.

## 2. Materials and Methods

### 2.1. Sample Selection

This research is a non-interventional, prospective, multicenter, post-marketing surveillance study carried out between August 2024 and August 2025 in 50 clinical centers specializing in the treatment of gastrointestinal problems across the country. During the study period, patients receiving trimebutine maleate as part of routine clinical care were screened for eligibility at participating centers. Consecutive adult patients who fulfilled the inclusion criteria and provided written informed consent were enrolled. Patients were excluded if they met any predefined exclusion criteria, including pregnancy, significant organ pathology, malignant gastrointestinal disease, or prior treatment with trimebutine. Patients with incomplete questionnaires or missing follow-up data were not included in the final analysis. Due to the non-interventional and observational nature of the study, formal documentation of all screened but non-enrolled patients was not mandatory at participating sites. A total of 2501 patients with complete baseline and follow-up assessments were included in the final study sample. Given the non-interventional design, patients were deemed appropriate for trimebutine at standard therapeutic doses according to their clinical profile and the particulars of their diagnosis and clinical severity of the identified condition, without their involvement in this study influencing the selection of a therapeutic strategy. Individuals aged 18 to 70 years, both male and female, with a confirmed diagnosis of IBS (irritable bowel syndrome) and/or functional dyspepsia, who have provided informed consent to participate in the study, are considered eligible for inclusion.

Diagnoses of functional gastrointestinal disorders have been made by gastroenterologists in accordance with standard clinical practice aligned with Rome IV criteria. Nonetheless, formal structured Rome IV questionnaires were not consistently utilized within the observational protocol. The study represents actual diagnostic decision-making in practice rather than reclassification in controlled trials.

Trimebutine is recognized in clinical practice as a regulatory agent targeting gastrointestinal motility and visceral hypersensitivity, particularly in patients with IBS and functional dyspepsia, where symptom-based treatment strategies are recommended [8,9].

### 2.2. Measurement Instruments

- *Izumo scale—primary outcome (global symptom severity and quality of life (QoL))*

The patient-reported therapeutic outcomes were derived from a pre-validated self-assessment questionnaire adapted from the Izumo scale [10]. The Izumo questionnaire is a recognized standardized tool for evaluating the quality of life associated with gastrointestinal disorders. The method has been substantiated by its use in several prior studies and is endorsed in the 2021 Japanese recommendations for functional dyspepsia as a legitimate self-assessment instrument for initial diagnosis and assessment of therapy efficacy in enhancing functional dyspepsia. The questionnaire comprises 15 items over 5 domains: reflux (items 1–3), pain (items 4–6), fullness (items 7–9), constipation (items 10–12), and diarrhea (items 13–15), with 3 items allocated to each category. Each question is evaluated using a 6-point Likert scale from 0 to 5, representing the patient’s subjective assessment of symptom severity, ranging from absence of symptoms to mild symptoms without necessitating a decline in quality of life to highly distressing levels of reported complaints. The comprehensive evaluation of each participant’s condition spans from 0 to 75 points, with each category allocated a score between 0 and 15 points. A lower score indicates less symptom severity. Furthermore, the most disturbing symptom for patients is selected as gastrointestinal symptoms that cause moderate to severe disruption in daily activities for each individual (a combination of symptoms may be specified if multiple illnesses are perceived as equally unpleasant by the respondent). The questionnaire includes sections for evaluating symptom severity prior to and following therapy, together with data on adverse drug reactions (ADRs).

- *Likert scale—grading symptom intensity over time*

A 6-point Likert scale (ranging from 0 = no symptoms to 5 = severe symptoms with substantial impact on daily activities) was used to quantify the intensity of individual symptoms over time. This scale enabled sensitive detection of changes in symptom severity between study visits and facilitated longitudinal comparison of patient-reported outcomes during trimebutine maleate treatment [11].

- *Bristol Stool Form scale—stool consistency/bowel habit classification*

The Bristol Stool Form Scale was applied to classify stool consistency and identify abnormalities in bowel habits. This instrument was used to support the evaluation of functional defecation disorders by categorizing stool morphology into standardized types, thereby complementing symptom severity data related to constipation and diarrhea [12].

The combined use of these instruments allowed a comprehensive and structured assessment of patient-reported gastrointestinal symptoms and their evolution during therapy.

It was intentional to use these complementary tools: the Bristol Stool Form Scale ensured objective classification of abnormalities in bowel habits, the Likert scale allowed sensitive longitudinal grading of symptom intensity, and the Izumo-based questionnaire offered a multidimensional assessment of global symptom burden and quality of life. When used in tandem, these instruments reduced measurement bias and improved internal consistency in the assessment of symptoms.

### 2.3. Statistical Analysis

A statistical analysis of the acquired data was conducted utilizing the specialist software IBM SPSS Statistics V.30 [13]. Descriptive statistics were used to summarize demographic and clinical characteristics of the study population. Categorical variables are presented as absolute numbers and percentages, while continuous and ordinal variables are presented as means with standard deviations.

Given the ordinal nature of symptom severity scores derived from Likert-type scales and the non-normal distribution of the data, non-parametric statistical methods were applied. Changes in symptom severity across repeated assessments (visits 1, 2, and 3) were evaluated using Friedman’s test for related samples. When pairwise comparisons between visits were required, the Wilcoxon signed-rank test was used.

The choice of non-parametric tests was determined by the distributional properties of the gathered data and the ordinal nature of Likert-type responses. Given that many measures were collected from the same subjects throughout three visits, Friedman’s test was deemed suitable for identifying general temporal variations, whereas Wilcoxon signed-rank tests were utilized for subsequent pairwise comparisons. This analytical approach maintained methodological consistency with the features of the measuring scale and circumvented assumptions of normality.

Between-group comparisons between patients with and without *H. pylori* infection were summarized descriptively due to the observational design of the study. Differences in the distribution of categorical variables across visits were analyzed using the chi-square test.

All statistical tests were two-sided, and a *p*-value of <0.05 was considered statistically significant.

The Ethics Committee for Scientific Research at VITA Hospital, Sofia, Bulgaria, examined and approved this study (statement number: 04-H/2024).

## 3. Results

The Izumo Scale was developed and validated as a questionnaire to evaluate the influence of gastrointestinal symptoms on quality of life over the preceding 7 days. Furthermore, the symptom that caused the greatest distress to patients was found to be gastrointestinal problems, which significantly interfered with daily activities for each individual (a combination of symptoms may be indicated if many disorders are perceived as equally unpleasant by the respondent). Unusual bowel movements were characterized as occurring more than three times per day or fewer than three times per week. The stool consistency, evaluated via the Bristol Stool Scale (Table 1) for stool morphology (types 1, 2, 6, or 7), was also recognized for diagnosing irregular bowel movements.

**Figure 1:**
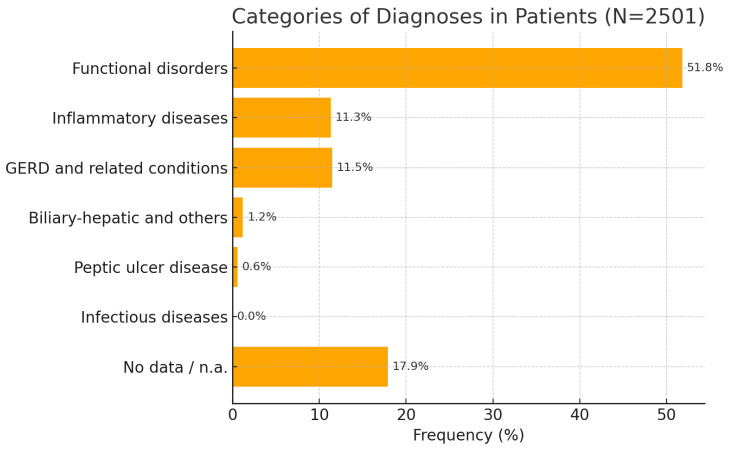
Classification of trial participants based on the primary diagnosis at the initiation of treatment with trimebutine maleate.

A total of 2501 patients were included in the analysis. The mean age of the participants was 46.8 years (SD 15.3). The study sample had about equal numbers of both sexes, with a marginally greater proportion of women (58.3%) compared to men (41.2%). The predominant symptoms are classified into several primary categories: “gastrointestinal discomfort” (87.4%), peristalsis disorders (51.6%), and dyspepsia (35.9%) (the cumulative percentage exceeds 100% as some respondents reported multiple leading symptoms). An exhaustive examination of the subcategories of primary diagnoses reported by patients seeking therapy reveals that the recognized nosological entities are irritable bowel syndrome (37.3%), gastritis (11.8%), gastroesophageal reflux disease (10.3%), and functional dyspepsia (7.2%), among others. For the summary analysis of the acquired data, the diagnoses reported by clinicians, in accordance with the International Classification of Diseases, 10th revision, were categorized into several primary groupings. The data analysis indicates that the predominant diagnoses are functional gastrointestinal illnesses, comprising 51.8% of all cases (Figure 1). Irritable bowel syndrome (IBS) is the predominant diagnosis, affecting 37.5% of patients, followed by the principal diagnoses related to treatment using trimebutine maleate:

- Gastroesophageal reflux disease and associated diseases—11.5%;
- Gastrointestinal tract inflammatory disorders—11.3%
- Biliary and hepatic diseases, along with structural abnormalities, account for 1.2%;
- Prevalence of peptic ulcer disease—0.6%;
- Intestinal infectious illnesses—0.0% (just one case documented)

In 17.9% of patients, the diagnosis was either unclear or required further elucidation during empirical symptomatic treatment with trimebutine for the primary symptoms.

**Figure 2:**
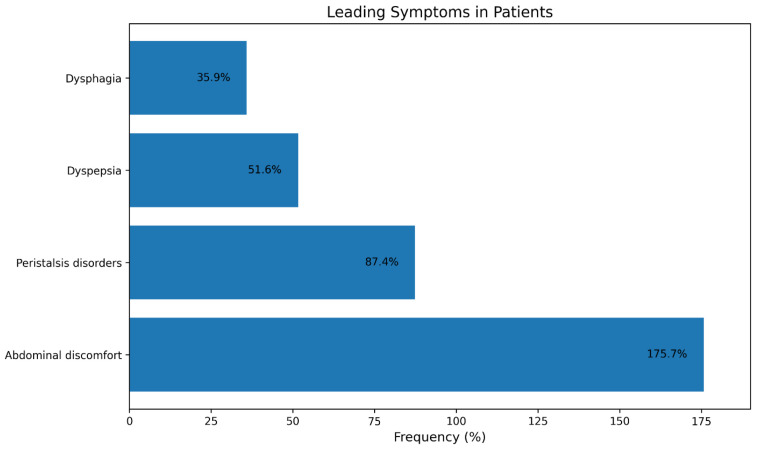
Distribution of primary symptoms described by patients as a condition for pursuing medical assistance and initiating treatment.

The predominant symptoms identified by participants as the primary reason for seeking a therapeutic-diagnostic consultation and requesting treatment include (Figure 2):

- Gastric distress—175.7% (The cumulative percentage surpasses 100% due to a considerable number of patients indicating multiple symptoms.)
- Disorders of peristalsis—87.4%
- Dyspepsia—51.6%
- Dysphagia—35.9%.

This pattern of complaints is characteristic of functional and chronic-recurrent gastrointestinal disorders. Abdominal discomfort and dyspepsia are primary symptoms frequently unlinked to conventional organic pathology, instead relating to motility abnormalities or visceral sensitivity issues.

The statistics underscore the necessity for a holistic and interdisciplinary strategy in the diagnosis and management of functional gastrointestinal diseases, which constitute the primary challenge in outpatient care.

The data produced during the current study indicate a distinct favorable trend in the clinical response to trimebutine maleate therapy regarding the alleviation of gastrointestinal pain symptoms (Table 1). In the context of the analysis, significant improvement is characterized by a decrease in pain to grade 0 or 1 following treatment. Approximately 91.05% (*n* = 2277) of patients see a notable decrease in abdominal pain severity relative to baseline following trimebutine medication.

**Figure 3:**
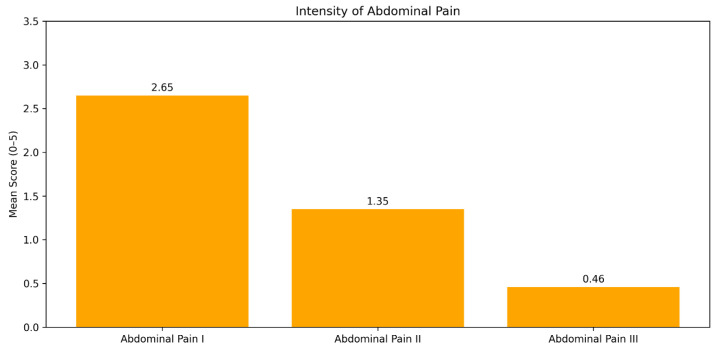
Average pain score documented at each study visit during treatment with trimebutine maleate.

Figure 3 and Table 2 illustrate the percentage distribution of abdominal pain severity scores in patients assessed at three consecutive visits: at the initiation of treatment (visit I), during treatment (visit II), and at the final evaluation of trimebutine therapy (visit III). Pain was evaluated using a 6-point scale (0—no pain, 5—maximum severity of pain). The percentage of patients indicating no pain (score 0) rose from 25.7% *(n* = 643) prior to therapy to 67.8% (*n* = 1697) following the conclusion of treatment. The proportion of patients experiencing moderate to severe pain (scores 3–5) diminished from 56.02 (*n* = 1401) at visit I to merely 1.7% (*n* = 42) at visit III. This outcome demonstrates the significant effectiveness of trimebutine in managing functional abdominal discomfort.

**Figure 4:**
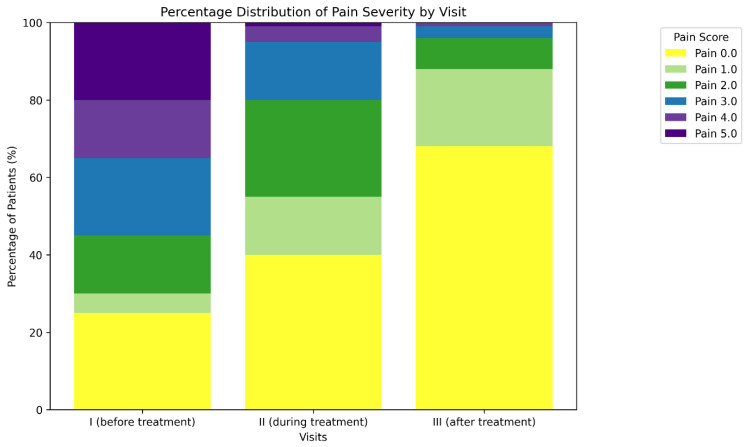
Patients’ subjective assessment of pain intensity, assessed from 0 to 5 at the time of prescription (visit 1) and during/after treatment with trimebutine maleate (visits 2/3).

The analysis of the correlation between abdominal pain, a principal symptom in the examined patients, and treatment with trimebutine maleate indicates a statistically significant enhancement post-therapy (Friedman χ^2^ = 244.512, *p* < 0.001). A statistically significant decrease in the severity of reported pain and burning sensation in the epigastric region was observed following treatment with trimebutine maleate, with mean scores of 2.65 (SD 1.711) at visit 1, 1.35 (SD 1.319) at visit 2, and 0.46 (SD 0.624) at the third examination of the participants, *p* < 0.001 (Figure 3). Figure 4 illustrates the dynamics of the average evaluation of abdominal discomfort during therapy with trimebutine maleate.

### 3.1. Dynamics of Gastroesophageal Reflux Manifestations

**Figure 5:**
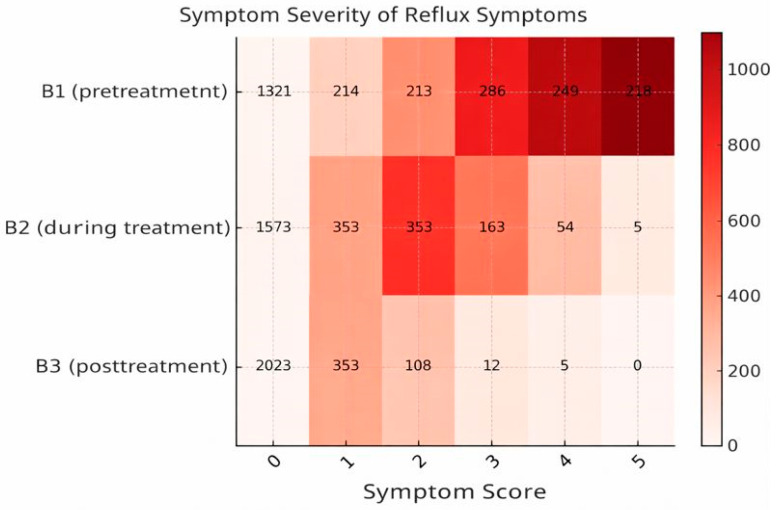
Assessment dynamics of GERD and heartburn symptoms during therapy with trimebutine maleate in the investigated sample.

Figure 5 illustrates the patterns of subjective evaluations of heartburn and reflux reported by individuals undergoing therapy with trimebutine maleate. The variation in the intensity of heartburn linked to upper dyspepsia symptoms, as described by patients, is again assessed using a 6-point Likert scale (0—no symptoms, 5—distressing symptoms with a substantial impact on quality of life).

The administration of trimebutine maleate therapy resulted in a notable enhancement in dyspeptic symptoms (Pearson χ^2^ test for distribution differences across visits: χ^2^ = 1317.18, *p* < 0.0001): the percentage of patients exhibiting no symptoms (score 0) rose from 52.8% (*n* = 1321) prior to treatment to 80.9% (*n* = 2023) following the conclusion of therapy. The proportion of patients with serious symptoms (scores 4 and 5) diminished from 18.6% (*n* = 467) at V11 to under 0.2% (*n* = 5) at V3 (Table 3).

**Figure 6:**
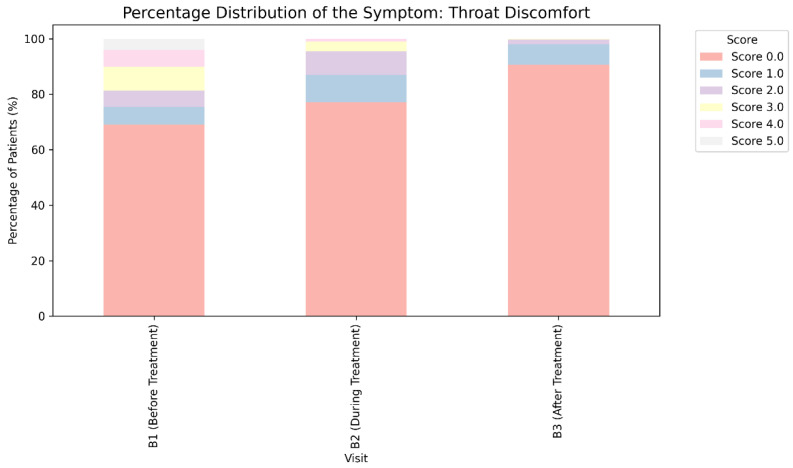
Change in the evaluation of the severity of the symptom “throat discomfort” linked to dyspepsia during treatment with trimebutine maleate in the examined patients.

**Figure 7:**
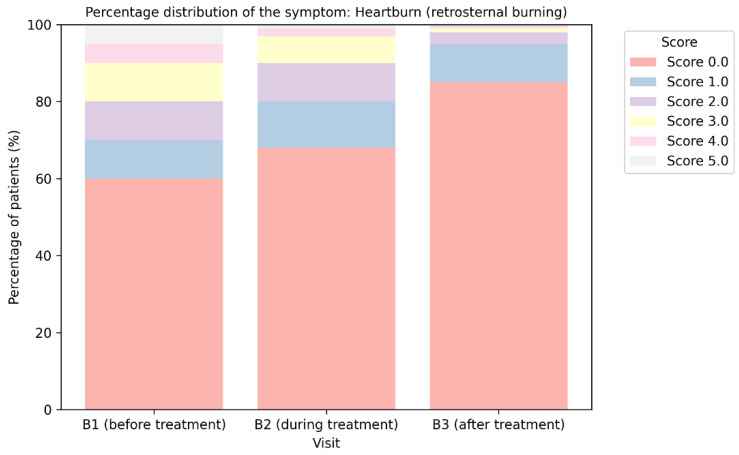
Change in participants’ evaluation of the intensity of the symptom “burning behind the breastbone” during therapy with trimebutine maleate among the examined patients.

The Izumo questionnaire was altered for this study to achieve comprehensive coverage of patient-reported effects on numerous symptoms, including those related to the same ailment or nosological unit. To mitigate the risk of distorting the derived conclusions, the trial participants also expressed their own perspectives on the relevance of two supplementary complaints associated with possible upper dyspeptic syndrome—burning behind the sternum and soreness in the throat. The analysis findings based on these criteria are depicted in Figure 6 and Figure 7.

During the third visit, 96.2% (*n* = 2124 + 282 = 2406) of patients indicated no or minimal burning sensation (scoring 0 or 1) behind the sternum. At baseline, prior to the initiation of treatment with trimebutine maleate (B1), the percentage was around 65.0% (*n* = 1454 + 170 = 1624), with a notable number of subjects exhibiting scores between 3 and 5 (Figure 7).

A comparable tendency was noted for problems characterized by patients as throat discomfort linked to upper dyspeptic syndrome. An increase in the relative proportion of participants reporting no symptoms or mild throat irritation owing to dyspepsia was observed with greater exposure to trimebutine maleate, rising from 75.49% (*n* = 1887 calculated as 1727 with score 0 plus 160 with score 1) at B1 to 97.96% (*n* = 2450 as 2268 with score 0 plus 182 with score 1) at visit 3 (Pearson χ^2^ test: χ^2^ = 692.83, *p* < 0.001). A notable enhancement in acid mouth irritation was distinctly observed as early as Visit 2 (Figure 6) (Table 4).

**Figure 8:**
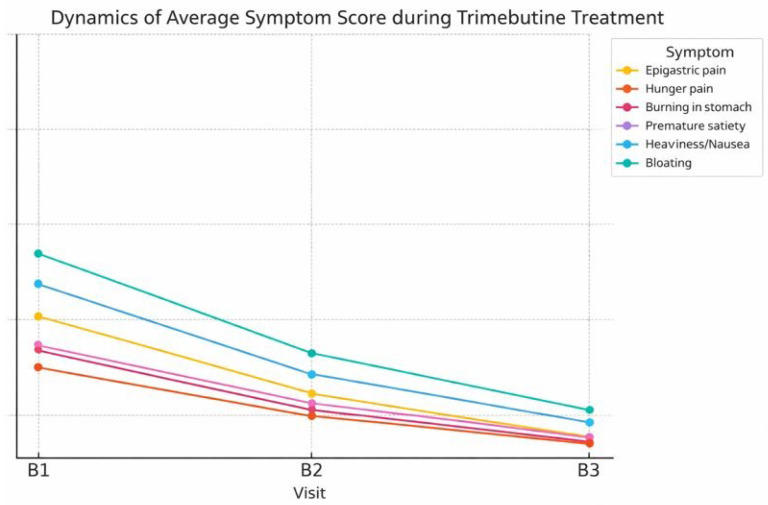
Dynamics in the average intensity of symptoms related to functional dyspepsia with therapy with trimebutine maleate in the individuals studied.

Figure 8 illustrates the changes in the mean score of the primary symptoms related to functional dyspepsia, observed during the therapy with trimebutine maleate. A notable decrease was observed in all assessed symptoms (Friedman test, *p* < 0.001). The most elevated starting values were recorded for symptoms of abdominal bloating (2.13) and sensations of heaviness/nausea (1.79). The symptoms exhibited the most substantial decrease in the average severity score post-treatment, reducing to 0.34 and 0.27, respectively. All clinical manifestations of functional dyspepsia exhibited a tendency towards values below 0.3 post-treatment, signifying nearly complete alleviation of subjective problems in the instances treated with trimebutine maleate.

**Figure 9:**
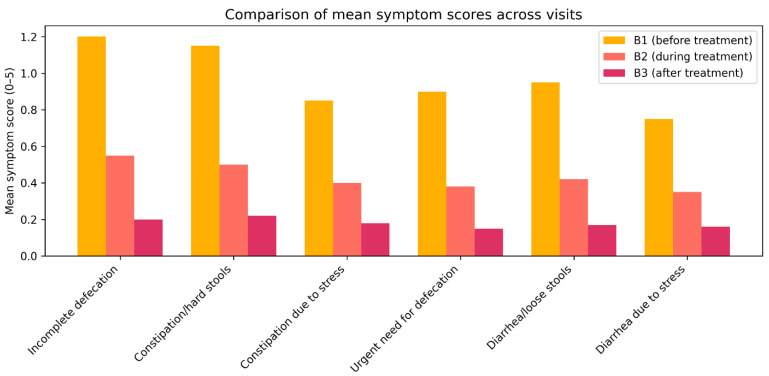
Variations in the mean score for symptom severity related to defecation anomalies between baseline and visit 3 of the trimebutine treatment period.

The current study reveals a distinct trend indicating a decrease in the average severity of symptoms across all categories of functional defecation disorders, with the average evaluation of complaints associated with constipation, diarrhea, stress-induced alterations, and sensations of incomplete defecation diminishing by 60–85% by visit B2 (Figure 9). At visit 3, study participants indicated that, while undergoing treatment with trimebutine maleate, the aforementioned symptoms associated with defecation regularity were nearly entirely managed, with Izumo severity scores approximately at or below 0.2 (indicating minimal or absent complaints). A significant trend is evident in the enhancement of the sensation of incomplete defecation (reduction from 1.26 to 0.22), alongside the categories of constipation and diarrhea, which both exhibit values below 0.2 post-treatment.

**Figure 10:**
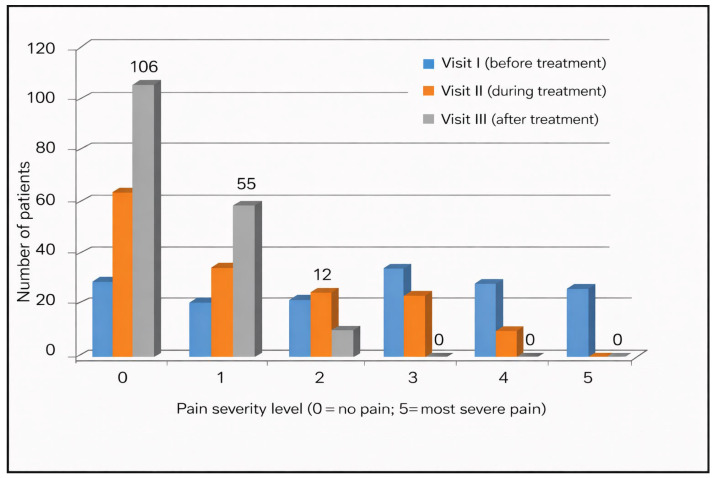
Distribution of abdominal pain severity scores in patients with confirmed *H. pylori* infection across Visits I–III.

**Figure 11:**
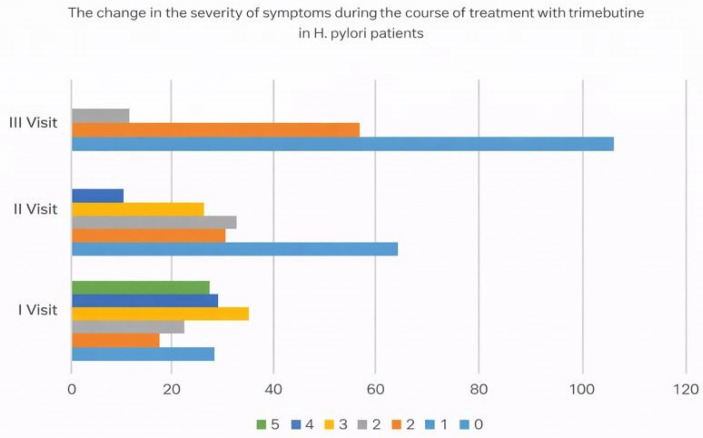
Distribution of symptom severity categories among *H. pylori*-positive patients during trimebutine maleate treatment.

**Figure 12:**
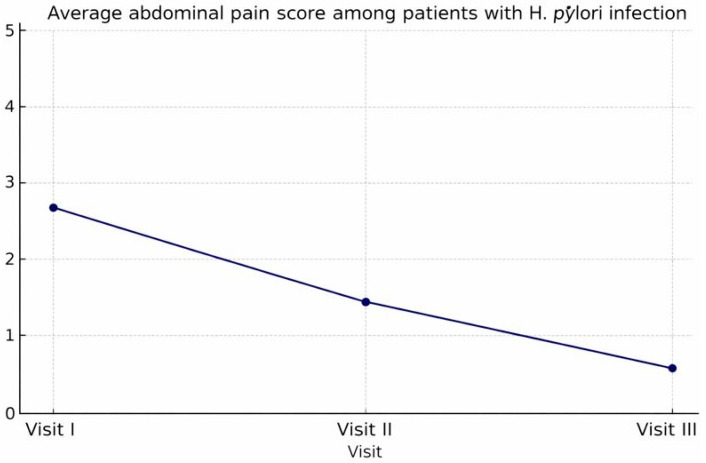
Mean abdominal pain severity score (0–5) in patients with confirmed Helicobacter pylori infection across Visits I–III. Changes across visits were assessed using Friedman’s test for repeated measures (*p* < 0.001).

An additional investigation was conducted on a subset of patients with proven Helicobacter pylori infection (*n* = 173). The mean pain score in the abdomen region shows a distinct trend of reduction with the advancement of trimebutine maleate treatment. The average symptom severity score reduced from a baseline value of 2.65 (visit 1) to 1.35 (visit 2), and by visit 3, patients reported an average symptom intensity of merely 0.46. The significant reduction in subjective pain feelings serves as a valid foundation for the anticipated high efficacy of trimebutine maleate treatment in patients with *H. pylori*, who often demonstrate a more severe clinical presentation of gastrointestinal discomfort (Figure 10, Figure 11 and Figure 12).

#### Safety Evaluation

Study participants have been requested to disclose any adverse effects experienced at each visit. No major adverse medication responses were reported by the study patients during the trial period. Patients were consistently questioned at each appointment regarding the emergence of new or exacerbated symptoms potentially associated with treatment. Mild and temporary symptoms, such as nausea or headache, were recorded in a tiny percentage of cases (*n* = 32, 1.27%) and have been assessed by the investigators as not clinically significant and not necessitating the discontinuation of medication. No cardiovascular or neurological adverse effects were recorded at any visit throughout this research. Nonetheless, the practical design and the corresponding absence of interventions for the clinical evaluation of designated biomarkers classify this study primarily as a means to assess the tolerability rather than the safety of trimebutine malate.

## 4. Discussion

Extensive systematic reviews and meta-analyses indicate that functional gastrointestinal problems are prevalent worldwide, impacting 15–25% of the population in affluent nations, with a notable tendency towards the “rejuvenation” of the condition [14,15].

The reliance on patient-reported outcomes in this study conforms to current methodological guidelines in functional gastrointestinal research, where symptom-based evaluation is the most clinically pertinent indicator of therapy efficacy. In the lack of dependable biomarkers, PRO tools continue to be fundamental for assessing treatment efficacy in FGIDs [9,16].

The analyzed sample demographically aligns with the target patients identified in prior research regarding treatment with trimebutine maleate and gastrointestinal functional problems, namely comprising predominantly working-age individuals (mean age 46.8) with a slight female predominance [17].

The clinical importance of gastrointestinal tract functioning disorders mostly arises from their substantial impact on the quality of life of affected individuals. The decline in the overall evaluation of quality-of-life results from the deterioration of physical health and the adverse emotional and psychological variables linked to functional gastrointestinal problems. The prevalent comorbidity of anxiety and depressive disorders in this patient population must not be overlooked. Consequently, consistent with prior research, the current trial deems it pertinent to evaluate the efficacy and safety of trimebutine from the standpoint of treated patients, particularly regarding their subjective assessment of quality of life, based on critical symptoms analyzed pre- and post-therapy with trimebutine maleate [18,19].

Moreover, research indicates compromised adherence in patients with functional gastrointestinal disorders, frequently due to polypharmacy, including pharmacological treatments involving large dosages and extended usage of medications affecting the gastrointestinal tract [20]. The patient’s viewpoint and subjective evaluation of the correlation between symptom alterations and trimebutine treatment are crucial for attaining the best health outcomes in individuals with functional gastrointestinal disorders.

The clinical profile of affected patients identified in this analysis predominantly exhibited a multisymptomatic presentation with prominent functional disorders, a slight female predominance, and moderate yet distinctly recognized pain symptoms, aligning with previously published epidemiological data on gastrointestinal functional disorders [21,22].

The findings from this study involving 2501 patients with functional gastrointestinal problems indicate significant clinical enhancement after treatment with trimebutine maleate. Substantial decreases in symptom severity, evaluated using the Likert scale, were noted for both pain and dyspeptic issues, as well as for defecation difficulties. More than 91% of patients indicated substantial improvement or total resolution of stomach discomfort post-treatment, a result corroborated by quantitative evaluations (average score drop from 2.13 to 0.30). A prospective multicenter trial by Maev et al. showed analogous findings, indicating that 74% of patients had alleviation of dyspeptic and peristaltic symptoms related to functional diseases, including GERD and IBS [21]. A comparable trend was observed in gastroesophageal reflux symptoms, as our data indicate that the proportion of asymptomatic patients (score 0) increased from 52.8% to 80.9% by the third visit. In the current investigation, patients exhibiting significant dyspeptic complaints (scores 4–5) diminished to below 0.2%. Kountouras et al. similarly discovered that the combination of trimebutine and proton pump inhibitors resulted in enhancements in gastroesophageal reflux and irritable bowel symptoms [23].

The fluctuations in the average intensity of functional dyspepsia, particularly evident in sensations like bloating and heaviness (declining from 2.13 and 1.79 to 0.34 and 0.27, respectively), substantiate the involvement of trimebutine in modulating intestinal motility and visceral sensitivity. A comparable theory is corroborated by published pharmacological findings about trimebutine’s capacity to influence enteric nervous system activity via partial inhibition of opioid receptors and motilin-regulating mechanisms [5,7].

The current analysis indicates that treatment with trimebutine results in comparable improvements in defecation disorders, including both constipation and diarrhea. Symptoms such as “feeling of incomplete defecation,” “stress-induced constipation,” and “diarrhea” exhibited a decrease in the average score to below 0.2 following treatment with trimebutine maleate. This aligns with findings from prior studies [6], including research, which indicated that trimebutine enhanced the frequency and consistency of defecation in individuals with chronic constipation [24].

Patients with proven Helicobacter pylori infection exhibited increased baseline pain severity (2.65) and a more significant reduction post-therapy, decreasing to 0.46 by visit 3. This corroborates the concept that trimebutine may be efficacious even in individuals with organic *H. pillory* superinfection, who are presumed to exhibit heightened visceral hypersensitivity [23].

This study lacked a systematic methodology to assess tolerability and safety through the identification of adverse drug reaction signals; however, patients did not express any concerns about the safe administration of trimebutine maleate during their visits, nor was there any indication of a causal link between adverse events and the use of the study product for the study period being assessed. Published literature presents data on minor, temporary gastrointestinal symptoms like nausea, headache, and alterations in peristalsis, which aligns with findings from a systematic review regarding the safety of prokinetic medications overall. Another noteworthy point is that the aforementioned discomforts may not be direct adverse effects of the administered trimebutine but may be linked to the pathophysiological repercussions of the gastrointestinal tract’s functioning disorders. It has been noted that headaches and the concomitant deterioration of quality of life may be linked to functional dyspepsia [25,26].

Our observations corroborate the safety of trimebutine, as evidenced by the findings of a systematic review [27], which assesses the tolerance of prokinetics in individuals with functional gastrointestinal problems. In the realm of functional dyspepsia, findings from a Bayesian meta-analysis indicate that trimebutine is considered one of the effective therapy alternatives [8]. Comparative studies also highlight the good tolerability of trimebutine in the context of other prokinetics, with no serious adverse reactions typical of dopaminergic antagonists (e.g., extrapyramidal effects) reported [27]. In the network meta-analysis conducted by Yang et al., trimebutine is positioned second in efficacy among prokinetics for functional dyspepsia, achieving a substantial SUCRA score of 74.5% [8].

The findings validate the significant effectiveness of trimebutine maleate in treating individuals with upper dyspeptic symptoms. The mean symptom score diminished by over 80–90% as early as visit B3, indicating a swift and enduring clinical response. The results acquired align with existing literature regarding the beneficial impact of combination therapy in functional dyspepsia associated with *H. pylori* [23]. Of interest is the comparison between the average assessment of abdominal pain in the general study population and in the subgroup of patients with proven *H. pylori* infection. At the beginning of the observation, patients with *H. pylori* reported a significantly higher subjective pain score (2.65 vs. 2.13), which is consistent with literature data describing increased nociceptive sensitivity and inflammatory activity in this group. Although both groups showed clear improvement with progressing treatment, the subgroup with *H. pylori* retained slightly higher residual values until the end of therapy (0.46 vs. 0.30). This can be explained both by the need for combined anti-Helicobacter therapy and by the potential role of *H. pylori* in persistent functional disorders, even after eradication. The statistics underscore the necessity for a comprehensive strategy in managing individuals with stomach pain and confirmed *H. pylori* infection.

**Figure 13:**
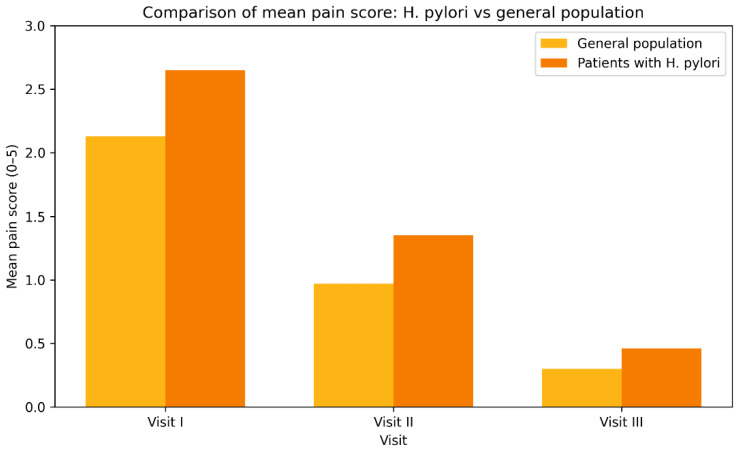
Comparison of mean abdominal pain severity scores (0–5) between the overall study population and patients with confirmed *Helicobacter pylori* infection across study visits. Results are presented descriptively.

Patients with confirmed *H. pylori* infection exhibited higher baseline abdominal pain severity compared with the overall study population (mean score 2.65 vs. 2.13). Although both groups demonstrated marked improvement during treatment with trimebutine maleate, residual symptom scores at Visit III remained slightly higher in the *H. pylori* subgroup (0.46 vs. 0.30). This finding aligns with the data from Kountouras et al., which indicates that the incorporation of trimebutine into proton-pump inhibitor (PPI) therapy for patients with GERD and IBS results in superior symptom management compared to monotherapy (Figure 13 and Table 5) [23].

Clinical relevance and implications for practice

This comprehensive prospective study enables us to derive several key implications relevant to clinical practice:

- The utilization of patent-reported outcomes exemplifies the individual patient-centered methodology used in current clinical and therapeutic practice.
- The initial therapeutic impact, reported at the second visit, is associated with enhanced patient adherence, hence diminishing the necessity for polypharmacy.
- The findings clearly demonstrate that trimebutine may serve as either a first-line or alternative treatment for people with irritable bowel syndrome characterized by predominant pain, individuals with concurrent gastroenterological disorders, and those affected by *H. pylori* and significant visceral hypersensitivity.

Strengths of the study

Our study has several significant strengths: a substantial sample size (*n* = 2501), a multicenter design (including 50 centers), and a prospective methodology. The utilization of a validated questionnaire derived from the Izumo scale guarantees methodological consistency in evaluating the data reported by the observed patients. A markedly heterogeneous outpatient group was included, enhancing the external validity of the results obtained. The distinct assessment of the subgroup of patients with proven Helicobacter pylori infection provides supplementary clinically relevant data, which is rarely noted in such observational research.

Study limitations

Notwithstanding the significant clinical usefulness of the findings, certain methodological constraints warrant acknowledgment. This research relies on data produced by a validated self-assessment questionnaire regarding the intensity of symptoms associated with functional gastrointestinal diseases. Nonetheless, the study is constrained by limitations commonly associated with patient-reported treatment results, including the potential for inadequate objectivity in clinical status evaluation and the risks of recall bias and expectancy effects. The current analysis lacks a control group for direct comparison, and the patient’s evaluation relies on subjective opinions and perceptions regarding functional problems and their related effects. The lack of a control group constitutes a significant methodological constraint. Functional gastrointestinal illnesses exhibit significant placebo response rates, often ranging from 30% to 40% in randomized therapeutic trials [28]. Consequently, while the extent and reliability of symptom enhancement seen in the current group are clinically significant, the possibility of a placebo effect cannot be dismissed. Future randomized controlled trials are necessary to validate the causal link between trimebutine treatment and the reported symptomatology. The analysis and interpretation of the data considered these limits, which were obtained by reputable statistical methods. Notwithstanding the constraints of the direct patient reporting technique, the investigation of patient experiences with trimebutine serves as a foundation for a comprehensive evaluation of compliance and related adherence to trimebutine therapy.

The diversity of underlying diagnoses constitutes a fundamental shortcoming of the current real-world observational design. Trimebutine was provided in accordance with standard clinical practice, focusing on symptom complexes rather than precisely disease-specific treatment protocols. Consequently, the findings should be understood as indicative of symptomatic enhancement across functional gastrointestinal manifestations rather than as evidence of disease-modifying effectiveness within certain diagnostic categories.

## 5. Conclusions

The investigation of patient experiences with trimebutine maleate reveals favorable evaluations from participants for its therapeutic efficacy as a conventional treatment for functional gastrointestinal problems. The study presents concrete evidence of substantial enhancement in primary symptoms, such as pain, bloating, heartburn, and defecation difficulties, as reported directly by patients. More than 90% of patients indicated a substantial decrease in symptoms post-treatment, with no severe adverse medication responses noted. The findings endorse the therapeutic application of trimebutine as the preferred medication for individuals with functional disorders and underscore the necessity for further randomized controlled trials to corroborate these results in more extensive populations and subgroups, including those infected with *H. pylori*. This study validates the significance of trimebutine in personalized and symptom-focused treatment for gastrointestinal functional disorders.

## Figures and Tables

**Table 1:** The Bristol Stool Chart. Reproduced from Wikimedia Commons under the Creative Commons Attribution-Share Alike 3.0 Unported License (CC BY-SA 3.0) and based on The Simplified Bristol Stool Form Scale by Lewis SJ, et al. 1997 [12].

Bristol Stool Chart			
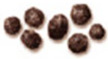	Type 1	Separate hard lumps	Very constipated
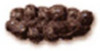	Type 2	Lumpy and sausage like	Slightly constipated
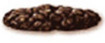	Type 3	A sausage shape with cracks in the surface	Normal
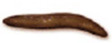	Type 4	Like a smooth, soft sausage or snake	Normal
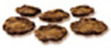	Type 5	Soft blobs with clear-cut edges	Lacking fibre
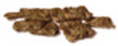	Type 6	Mushy consistency with ragged edges	Inflammation
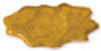	Type 7	Liquid consistency with no solid pieces	Inflammation

**Table 2:** Distribution of patients according to abdominal pain severity scores (0–5) at baseline (Visit I), during treatment (Visit II), and after treatment (Visit III). Changes across visits were analyzed using Friedman’s test for repeated measures, followed by Wilcoxon signed-rank tests for pairwise comparisons.

Severity	0	1	2	3	4	5	Total:
Visit I	643	127	330	619	493	289	2501
Visit II	1040	460	564	327	99	11	2501
Visit III	1697	581	181	33	7	2	2501

**Table 3:** Distribution of patients by severity of heartburn symptoms (scores 0–5) assessed at Visits I, II, and III. Differences in symptom severity distributions across visits were evaluated using the chi-square (χ^2^) test.

	0	1	2	3	4	5
	Count	Count	Count	Count	Count	Count
To what extent do heartburn symptoms disturb you? V1	1321	214	213	286	249	218
To what extent do heartburn symptoms disturb you? V2	1573	353	353	163	54	5
To what extent do heartburn symptoms disturb you? V3	2023	353	108	12	5	0

**Table 4:** Mean severity scores for symptoms associated with functional dyspepsia at baseline (Visit I), during treatment (Visit II), and after treatment (Visit III). Changes over time were analyzed using Friedman’s test for repeated measures.

Symptom	Mean Severity Assessment (0–5)		
	B1 (Pre-Treatment)	B2 (During Treatment)	B3 (Post-Treatment)
**Epigastral pain**	1.47	0.64	0.18
**Abdominal discomfort resembling hunger pangs**	0.91	0.42	0.11
**Stomach burning sensation**	1.11	0.49	0.14
**Premature satiety**	1.16	0.56	0.18
**Permanent sensation of abdominal heaviness/nausea**	1.79	0.81	0.27
**Stomach bloating**	2.13	1.00	0.34

**Table 5:** Comparison of mean abdominal pain severity scores between the general study population and patients with confirmed *Helicobacter pylori* infection across study visits. Results are presented descriptively.

Visit	General Study Population	*H. pylori* Subgroup
Visit I	2.13	2.65
Visit II	0.97	1.35
Visit III	0.30	0.46

## Data Availability

The original contributions presented in this study are included in the article. Further inquiries can be directed to the corresponding author.

## Abbreviations

The following abbreviations are used in this manuscript:

FGIDs	Functional gastrointestinal disorders
GERD	Gastroesophageal reflux disease
IBS	irritable bowel syndrome
PPI	Proton-pump inhibitor
ADR	Adverse drug reaction
SUCRA	Surface Under the Cumulative Ranking curve
